# Ultrasound-guided hydrodissection combined with acupotomy for moderate-to-severe Carpal tunnel syndrome: electrophysiological and ultrasonographic outcomes and their potential relationship to neuroinflammatory attenuation

**DOI:** 10.3389/fcell.2026.1930934

**Published:** 2026-09-09

**Authors:** Min Hu, Hongmei Huang, Binghang Zhu, Qing Xu, Zhubin Feng, Ye Xu, Juan Hu, Shiyu Chen

**Affiliations:** 1 Department of Ultrasonography, Yueqing Hospital of Wenzhou Medical University (People’s Hospital of Yueqing), Wenzhou City, Zhejiang, China; 2 Department of Ultrasonography, Ezhou Central Hospital, Ezhou City, Hubei, China

**Keywords:** acupotomy, carpal tunnel syndrome, electrophysiology, hydrodissection, minimally invasive treatment, nerve conduction, neuroinflammation, ultrasonography

## Abstract

**Background:**

Carpal tunnel syndrome (CTS) is a common compression neuropathy in which sustained median nerve ischemia drives progressive neuroinflammation, structural remodeling of the transverse carpal ligament (TCL), and functional nerve impairment. Current interventions typically address either the inflammatory or structural component of the disease, but rarely both simultaneously, and clinical evidence for combined approaches in moderate-to-severe CTS remains limited.

**Objective:**

To evaluate the clinical efficacy of ultrasound-guided hydrodissection combined with acupotomy in moderate-to-severe CTS, and to explore whether improvements in electrophysiological and ultrasonographic outcomes are consistent with attenuation of neuroinflammatory processes, while acknowledging that direct molecular confirmation was not performed in this study.

**Methods:**

This single-center retrospective cohort study enrolled 100 patients with moderate-to-severe CTS (February 2019–February 2025). The dual-axis group (n = 49) received hydrodissection combined with acupotomy; the single-axis group (n = 51) received hydrodissection alone. Primary outcomes were BCTQ-SSS and BCTQ-FSS scores; secondary outcomes included VAS, nerve electrophysiological biomarkers (SCV, DML, CMAP), ultrasonographic biomarkers (nerve CSA, TCL thickness), clinical efficacy grading, and adverse events at baseline, 1 week, 1 month, 3 months, and 6 months.

**Results:**

Both groups improved significantly from baseline (P < 0.05). The dual-axis group demonstrated significantly superior BCTQ-SSS, BCTQ-FSS, and VAS scores from 1 month onward (all Bonferroni-corrected P < 0.001). At 6 months, the dual-axis group showed markedly superior electrophysiological indicators of nerve functional recovery (SCV: 42.36 ± 4.87 vs. 38.92 ± 5.23 m/s; DML: 4.12 ± 0.65 vs. 4.58 ± 0.71 ms; CMAP: 7.85 ± 1.52 vs. 6.92 ± 1.64 mV; all P < 0.05) and greater reduction in ultrasonographic biomarkers of inflammatory edema (CSA: 10.35 ± 1.86 vs. 11.82 ± 2.14 mm^2^; TCL: 2.15 ± 0.38 vs. 2.68 ± 0.42 mm; all P < 0.001). The overall response rate was 93.9% vs. 80.4% (P = 0.031). No serious adverse events occurred.

**Conclusion:**

Ultrasound-guided hydrodissection combined with acupotomy demonstrated superior clinical, electrophysiological, and ultrasonographic outcomes compared with hydrodissection alone in moderate-to-severe CTS. Improvements in nerve conduction velocity, distal motor latency, CMAP amplitude, and median nerve cross-sectional area are consistent with, and may reflect, attenuation of underlying neuroinflammatory processes; however, direct molecular evidence for such a mechanistic connection was not obtained in this study and the association remains a hypothesis requiring prospective validation. These findings support the combined approach as a minimally invasive option for patients who are unable or unwilling to undergo surgical decompression, pending confirmation by prospective randomized trials with a surgical comparator arm.

## 1 Introduction

### 1.1 Cell death as a central driver of inflammatory disease

Chronic inflammatory diseases represent major global health burdens characterized by self-perpetuating cycles of tissue injury and immune dysregulation (Galluzzi et al., 2018; Bertheloot et al., 2021). Recent advances have revealed that distinct modes of regulated cell death—apoptosis, necroptosis, pyroptosis, and ferroptosis—release specific repertoires of damage-associated molecular patterns (DAMPs) that actively amplify and sustain inflammation (Galluzzi et al., 2018; Tang et al., 2019). Each modality engages different inflammatory circuits: failed apoptotic clearance exposes nuclear autoantigens; necroptosis releases HMGB1 and mitochondrial DNA through membrane rupture; pyroptosis drives gasdermin D (GSDMD) pore–mediated IL-1β and IL-18 secretion via inflammasome activation; and ferroptosis generates lipid peroxidation products activating innate immune sensors (Kelley et al., 2019; Shi et al., 2017; Weinlich et al., 2017). The resulting “death–inflammation–redeath” cycle has emerged as a unifying framework for understanding disease chronicity across diverse inflammatory conditions (Bertheloot et al., 2021; Tang et al., 2019).

Despite advances in delineating individual cell death pathways, a comprehensive understanding of how these modalities interact dynamically within complex inflammatory microenvironments remains incomplete (Galluzzi et al., 2018; Bertheloot et al., 2021; Tang et al., 2019). Critical questions persist regarding the molecular switch points dictating transitions between reversible inflammation and irreversible tissue destruction, and the identification of clinically accessible biomarkers reflecting cumulative cell death burden. Addressing these gaps is essential for developing precision-targeted therapies that disrupt pathogenic cell death–inflammation loops at multiple regulatory nodes.

### 1.2 Carpal tunnel syndrome as a clinical model of compression-driven inflammatory cell death

Carpal tunnel syndrome (CTS), the most prevalent peripheral nerve entrapment disorder affecting 1%–5% of the general population, provides a compelling clinical model for studying the interplay between mechanical stress, cell death, and neuroinflammation (Padua et al., 2023; Gebrye et al., 2024). CTS arises from compression of the median nerve within the rigid osteofibrous carpal tunnel, but its pathological progression extends beyond mechanical impingement—it is orchestrated by a self-amplifying cascade of ischemia-induced cell death and inflammatory amplification (Malakootian et al., 2023).

Chronically elevated intracarpal pressure impairs endoneurial microvascular perfusion, generating a hypoxic–ischemic microenvironment that drives mitochondrial dysfunction, reactive oxygen species (ROS) accumulation, and activation of intrinsic apoptotic pathways in Schwann cells and axons (Schmid et al., 2014; Gaudet et al., 2011). Schwann cell apoptosis—evidenced by cytochrome c release, caspase-9/caspase-3 activation, and Bcl-2 downregulation—dismantles myelin sheaths, producing the demyelination and axonal degeneration characteristic of CTS electrophysiology (Schmid et al., 2014). Concurrently, DAMPs released from dying cells—including HMGB1, S100 proteins, and extracellular ATP—activate pattern recognition receptors on resident macrophages and mast cells, triggering NLRP3 inflammasome assembly and gasdermin D–mediated pyroptotic release of IL-1β and IL-18 (Kelley et al., 2019; Shi et al., 2017; Lu et al., 2013). The resulting pro-inflammatory milieu sustains synovial hyperplasia, collagen deposition, and transverse carpal ligament (TCL) thickening, further elevating intracarpal pressure—closing a vicious “compression–ischemia–cell death–neuroinflammation–recompression” cycle (Malakootian et al., 2023; Moalem-Taylor et al., 2017; Baričić et al., 2022).

Inflammatory cytokines—TNF-α, IL-6, and IL-1β—are significantly elevated in CTS synovial tissue and patient serum, correlating with disease severity and serving as indirect biomarkers of ongoing cell death–driven inflammation (Moalem-Taylor et al., 2017; Baričić et al., 2022). HMGB1, a prototypical DAMP released from necroptotic and pyroptotic cells, perpetuates neuroinflammation through TLR4 and RAGE signaling (Feldman et al., 2012; Tang et al., 2012). This multi-modal cell death landscape makes CTS an ideal clinical context for evaluating precision interventions targeting multiple nodes of the cell death–inflammation axis.

### 1.3 Rationale for dual-axis precision targeting

Current CTS therapies address either the inflammatory or structural arm of the pathological cycle, but rarely both simultaneously (Ashworth et al., 2023; Graham et al., 2016). Ultrasound-guided carpal tunnel hydrodissection mechanically separates the median nerve from perineural adhesions while delivering corticosteroids that suppress NF-κB signaling, NLRP3 inflammasome priming, and pyroptotic IL-1β release (Kumar et al., 2023; Sveva et al., 2024; Lee et al., 2025). However, hydrodissection cannot address TCL-mediated mechanical compression, and its anti-inflammatory benefits wane as the corticosteroid is metabolized (Chan et al., 2025). Acupotomy, a minimally invasive blade-needle technique, achieves percutaneous TCL release under ultrasound guidance, substantially reducing intracarpal pressure and potentially attenuating the upstream ischemic stimulus for apoptotic cascades (Zhou et al., 2023; Chou et al., 2023; Sun et al., 2023), although the long-term durability of this structural change requires prospective validation beyond the 6-month window of the present study.

The combination constitutes a dual-axis precision strategy: hydrodissection targets the inflammatory–pyroptotic arm (downstream DAMPs and cytokine amplification), while acupotomy eliminates the structural–ischemic arm (upstream apoptotic trigger). This aligns with the emerging paradigm advocating simultaneous modulation of multiple cell death regulatory nodes for durable disease control (Bertheloot et al., 2021; Tang et al., 2019).

### 1.4 Study objectives

This retrospective cohort study aimed to: (1) evaluate the efficacy and safety of dual-axis intervention (hydrodissection + acupotomy) versus single-axis intervention (hydrodissection alone) in moderate-to-severe CTS; (2) characterize electrophysiological and ultrasonographic parameters as functional biomarkers of cell death resolution; and (3) provide translational evidence supporting precision multi-target modulation of cell death in compression-driven inflammatory neuropathy.

## 2 Materials and methods

### 2.1 Study design and ethical approval

This single-center retrospective cohort study collected clinical data from consecutive patients with moderate-to-severe CTS admitted between February 2019 and February 2025. The study was approved by the Medical Ethics Committee of Yueqing People’s Hospital, Wenzhou Medical University (Approval No. YQYY202400009), with waiver of informed consent due to the retrospective design. All procedures accorded with the Declaration of Helsinki.

### 2.2 Diagnostic criteria and severity stratification

CTS was diagnosed per the Chinese Expert Consensus on Diagnosis and Treatment of Carpal Tunnel Syndrome, requiring: (i) typical symptoms (nocturnal paresthesia, pain, numbness in the median nerve distribution; in severe cases, thenar atrophy); (ii) positive Phalen test and/or Tinel sign; (iii) electrophysiological confirmation of reduced SCV and/or prolonged DML. Disease severity was stratified as: Moderate—sensory conduction abnormalities with DML >4.5 ms, normal or mildly reduced CMAP; Severe—absent or markedly abnormal sensory conduction, markedly prolonged DML, markedly reduced or absent CMAP. This electrophysiological grading is consistent with, but does not directly measure, the cumulative burden of Schwann cell apoptosis and axonal degeneration.

### 2.3 Patient selection

Inclusion criteria:

(i) Confirmed moderate or severe CTS on electrophysiology; (ii) age 18–75 years; (iii) disease duration ≥3 months with inadequate response to conservative management; (iv) complete clinical data and follow-up ≥ 6 months.

Exclusion criteria:

Concurrent cervical spondylosis, diabetic peripheral neuropathy, or other conditions affecting upper limb nerve function; previous wrist surgery or fracture; space-occupying carpal tunnel lesions; pregnancy or lactation; severe cardiac, hepatic, or renal insufficiency; coagulation disorders; local infection; or allergy to study medications.

Based on treatment allocation, 49 patients constituted the dual-axis group (hydrodissection + acupotomy) and 51 the single-axis group (hydrodissection alone). Treatment allocation was determined by shared clinical decision-making between the treating physician and the patient, based primarily on patient preference regarding procedural invasiveness, the treating physician’s ultrasound-based assessment of transverse carpal ligament morphology, and patient-specific contraindications. This non-randomized allocation introduces potential clinician preference and referral bias that cannot be fully excluded despite *post hoc* baseline comparability.

### 2.4 Intervention protocols

#### 2.4.1 Hydrodissection: anti-inflammatory cell death suppression (both groups)

Patients were positioned supine with the affected limb abducted, wrist in slight dorsiflexion. Under aseptic conditions, a high-frequency linear array ultrasound probe (10–18 MHz) was placed transversely at the wrist crease. Using an in-plane technique with a 22G needle from the ulnar aspect, a 10 mL mixture (2% lidocaine 2 mL + compound betamethasone 1 mL + 0.9% saline 7 mL) was injected under real-time monitoring, ensuring circumferential fluid dissection. The corticosteroid targets the inflammatory–pyroptotic arm through NF-κB suppression, phospholipase A2 inhibition, and NLRP3 inflammasome priming attenuation (Ashworth et al., 2023; Kumar et al., 2023).

#### 2.4.2 Acupotomy: elimination of the upstream ischemic cell death trigger (dual-axis group only)

Following hydrodissection, a No. 4 disposable sterile acupotomy needle (blade width 0.4 mm, blade length 25 mm) was introduced under continuous ultrasound guidance to the TCL midpoint with the blade parallel to the median nerve. A longitudinal cutting technique performed 2–3 cuts along the nerve’s long axis with depth sufficient to penetrate the full TCL thickness. Intraoperative ultrasound confirmed disruption of TCL fibrillar continuity. All acupotomy procedures were performed by one of two senior ultrasonographists, each with a minimum of 8 years of experience in musculoskeletal ultrasound-guided intervention. Operators followed a standardized stepwise protocol requiring pre-specified intraoperative ultrasound confirmation of full-thickness TCL fibrillar discontinuity as a mandatory procedural endpoint. This intervention may substantially reduce intracarpal pressure and potentially restore endoneurial perfusion, thereby attenuating the upstream ischemic stimulus for ROS-mediated apoptotic signaling and DAMP release (Zhou et al., 2023; Chou et al., 2023; Sun et al., 2023); however, the long-term durability of this structural effect requires prospective validation beyond the 6-month follow-up reported here.

Postoperatively, both groups elevated the affected limb and avoided strenuous activity. Structured finger flexion–extension and median nerve gliding exercises began from day 2. NSAIDs were permitted for breakthrough pain.

### 2.5 Outcome measures

#### 2.5.1 Patient-reported biomarkers of neuroinflammatory cell death burden

BCTQ-SSS (11 items, 1–5 Likert) and FSS (8 items, 1–5 Likert) were assessed at baseline, 1 week, 1 month, 3 months, and 6 months. The VAS (0–10 cm) measured pain intensity as a readout of inflammatory nociceptive sensitization.

#### 2.5.2 Electrophysiological biomarkers of schwann cell death and axonal integrity

Median nerve SCV, DML, and CMAP amplitude were measured at baseline and 6 months. SCV reduction and DML prolongation reflect Schwann cell apoptosis–driven demyelination; reduced CMAP indicates axonal degeneration from cumulative apoptotic and necroptotic neuronal injury (Schmid et al., 2014; Koo et al., 2023).

#### 2.5.3 Ultrasonographic biomarkers of inflammatory edema and structural remodeling

Median nerve CSA at the carpal tunnel inlet and TCL thickness were measured at baseline and 6 months. Enlarged CSA reflects endoneurial edema sustained by inflammatory cell death products; its reduction indicates neuroinflammatory resolution (Savage and McKell, 2024). TCL thickness reduction reflects acupotomy-mediated ligament release (Sun et al., 2023).

#### 2.5.4 Clinical efficacy and safety

At 6 months: Excellent—BCTQ decrease ≥50%; Good—decrease 25%–49%; Poor—< 25% improvement or worsening. Overall response rate = (Excellent + Good)/total × 100%. Adverse events were recorded throughout follow-up.

### 2.6 Statistical analysis

Analyses were performed with SPSS 26.0. Continuous variables: mean ± SD or median (Q1, Q3). Categorical variables: n (%). Baseline comparisons: independent t-test, Mann–Whitney U, or chi-square. Longitudinal BCTQ/VAS: repeated-measures ANOVA with Bonferroni correction (threshold P < 0.0125). Electrophysiological/ultrasonographic comparisons: paired and independent t-tests. Efficacy grades: Mann–Whitney U. Adverse events: Fisher’s exact test. P < 0.05 was considered significant.

## 3 Results

### 3.1 Baseline comparability

No significant differences were observed between groups in sex, age, disease duration, BMI, comorbidities, occupation type, or electrophysiological severity grading (all P > 0.05; Table 1), confirming comparable baseline demographic and electrophysiological characteristics. Unmeasured confounders—including dominant hand status, baseline nerve atrophy grade, and patient preference for procedural invasiveness—may nonetheless have differed between groups and represent a potential source of selection bias.

**TABLE 1:** Baseline characteristics.

Parameter	Dual-axis (n = 49)	Single-axis (n = 51)	t/χ^2^/Z	P
Sex: Male/Female	14/35	13/38	0.125	0.724
Age (years, x̄ ± s)	52.3 ± 10.2	51.8 ± 9.7	0.254	0.800
Disease duration [M(Q1, Q3)]	14.0 (8.0, 24.0)	15.0 (9.0, 22.0)	−0.312	0.755
BMI (kg/m^2^)	25.1 ± 3.2	24.8 ± 3.4	0.461	0.646
Manual laborer [n (%)]	31 (63.3)	33 (64.7)	0.041	0.839
Moderate CTS [n (%)]	32 (65.3)	33 (64.7)	0.008	0.927
Hypertension [n (%)]	12 (24.5)	14 (27.5)	0.117	0.732
Diabetes [n (%)]	8 (16.3)	7 (13.7)	0.134	0.714
Hypothyroidism [n (%)]	5 (10.2)	6 (11.8)	0.064	0.800

### 3.2 Patient-reported biomarkers of neuroinflammatory burden

#### 3.2.1 Symptom severity scale (SSS)

Repeated-measures ANOVA: time F = 486.235, P < 0.001; group F = 12.847, P = 0.001; interaction F = 8.562, P < 0.001. Both groups improved significantly from baseline (P < 0.05). At 1 week, SSS scores were comparable (P > 0.0125). From 1 month onward, the dual-axis group showed significantly lower SSS (all P < 0.001; Table 2).

**TABLE 2:** SSS Scores (points, x̄ ± s).

Group	n	Baseline	1 Week	1 Month	3 Months	6 Months
Dual-axis	49	3.42 ± 0.58	2.31 ± 0.49	1.72 ± 0.41	1.48 ± 0.38	1.35 ± 0.32
Single-axis	51	3.38 ± 0.61	2.43 ± 0.52	2.05 ± 0.46	1.82 ± 0.43	1.71 ± 0.39
Corrected P	​	1.000	1.000	<0.001	<0.001	<0.001

#### 3.2.2 Functional status scale (FSS)

Time F = 412.786, P < 0.001; group F = 10.235, P = 0.002; interaction F = 6.874, P = 0.001. Significantly lower FSS in the dual-axis group from 1 month through 6 months (all P < 0.001; Table 3).

**TABLE 3:** FSS Scores (points, x̄ ± s).

Group	n	Baseline	1 Week	1 Month	3 Months	6 Months
Dual-axis	49	2.89 ± 0.52	2.15 ± 0.44	1.58 ± 0.36	1.38 ± 0.31	1.26 ± 0.28
Single-axis	51	2.85 ± 0.55	2.24 ± 0.47	1.86 ± 0.42	1.65 ± 0.38	1.54 ± 0.35
Corrected P	​	1.000	1.000	<0.001	<0.001	<0.001

#### 3.2.3 VAS pain scores

**FIGURE 1:**
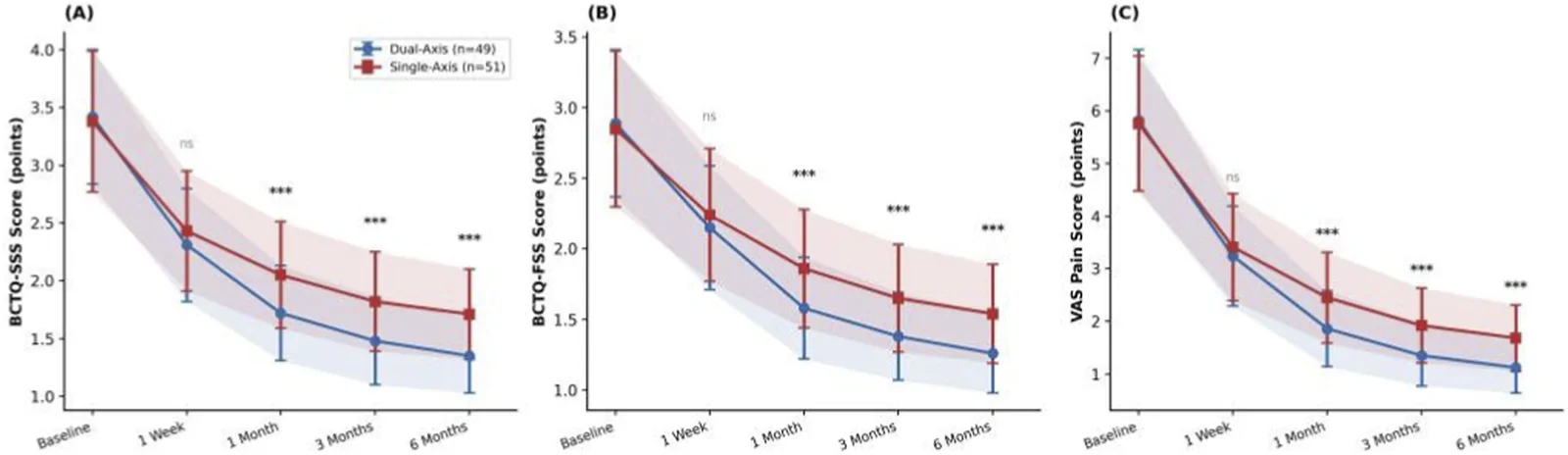
Longitudinal patient-reported outcome biomarkers over 6 months. **(A)** BCTQ-SSS, **(B)** BCTQ-FSS, and **(C)** VAS pain scores for the Dual-Axis group (blue, n = 49) and the Single-Axis group (red, n = 51). Data: mean ± SD; shaded bands: ±1 SD. Between-group comparisons: repeated-measures ANOVA with Bonferroni correction. ns, not significant at 1 week; ***P < 0.001 at 1, 3, and 6 months.

Time F = 523.412, P < 0.001; group F = 9.876, P = 0.002; interaction F = 7.235, P < 0.001. Non-significant at 1 week; significant from 1 month through 6 months (all P < 0.001; Table 4). The longitudinal changes in BCTQ- SSS, BCTQ-FSS, and VAS scores are shown in Figure 1.

**TABLE 4:** VAS pain Scores (points, x̄ ± s).

Group	n	Baseline	1 Week	1 Month	3 Months	6 Months
Dual-axis	49	5.82 ± 1.34	3.24 ± 0.95	1.86 ± 0.72	1.35 ± 0.58	1.12 ± 0.48
Single-axis	51	5.76 ± 1.28	3.41 ± 1.02	2.45 ± 0.86	1.92 ± 0.71	1.68 ± 0.63
Corrected P	​	1.000	1.000	<0.001	<0.001	<0.001

### 3.3 Electrophysiological parameters of nerve functional recovery

**FIGURE 2:**
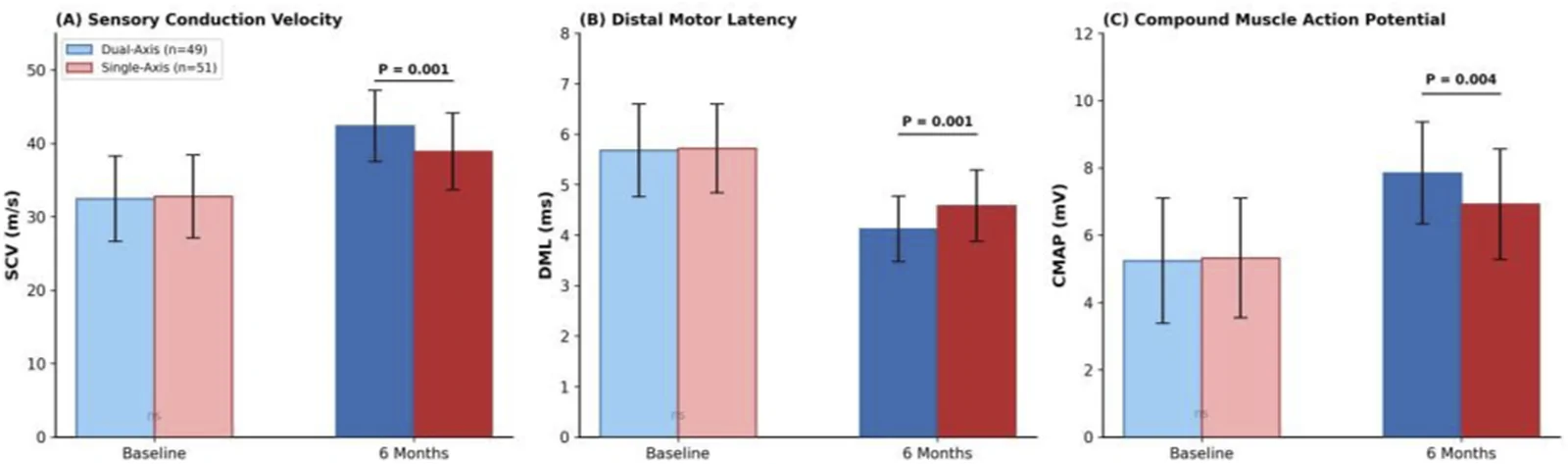
Electrophysiological biomarkers of cell death resolution at 6 months. **(A)** Sensory conduction velocity (SCV), **(B)** distal motor latency (DML), and **(C)** compound muscle action potential (CMAP) at baseline (light) and 6 months (dark) for the Dual-Axis (blue) and Single-Axis (red) groups. Error bars: ±1 SD. Higher SCV and CMAP indicate better nerve function; lower DML indicates improvement.

Baseline parameters were comparable (all P > 0.05). At 6 months, both groups improved significantly (all P < 0.001). The dual-axis group showed superior recovery: SCV 42.36 ± 4.87 vs. 38.92 ± 5.23 m/s (P = 0.001); DML 4.12 ± 0.65 vs. 4.58 ± 0.71 ms (P = 0.001); CMAP 7.85 ± 1.52 vs. 6.92 ± 1.64 mV (P = 0.004) (Table 5; Figure 2).

**TABLE 5:** Electrophysiological biomarkers (x̄ ± s).

Parameter	Time	Dual-axis (n = 49)	Single-axis (n = 51)	t	P
SCV (m/s)	Baseline	32.45 ± 5.82	32.78 ± 5.64	−0.291	0.772
	6 Months	42.36 ± 4.87	38.92 ± 5.23	3.432	0.001
DML (ms)	Baseline	5.68 ± 0.92	5.72 ± 0.88	−0.225	0.822
	6 Months	4.12 ± 0.65	4.58 ± 0.71	−3.408	0.001
CMAP (mV)	Baseline	5.24 ± 1.86	5.32 ± 1.78	−0.222	0.825
	6 Months	7.85 ± 1.52	6.92 ± 1.64	2.964	0.004

### 3.4 Ultrasonographic biomarkers of inflammatory remodeling

Baseline CSA and TCL thickness were comparable (P > 0.05). At 6 months, both groups showed significant reductions (all P < 0.001). The dual-axis group demonstrated significantly smaller CSA (10.35 ± 1.86 vs. 11.82 ± 2.14 mm^2^; P < 0.001) and TCL thickness (2.15 ± 0.38 vs. 2.68 ± 0.42 mm; P < 0.001) (Table 6).

**TABLE 6:** Ultrasonographic biomarkers (x̄ ± s).

Parameter	Time	Dual-axis (n = 49)	Single-axis (n = 51)	t	P
CSA (mm^2^)	Baseline	15.62 ± 2.84	15.48 ± 2.76	0.254	0.800
	6 Months	10.35 ± 1.86	11.82 ± 2.14	−3.694	<0.001
TCL (mm)	Baseline	3.28 ± 0.52	3.24 ± 0.48	0.405	0.686
	6 Months	2.15 ± 0.38	2.68 ± 0.42	−6.687	<0.001

### 3.5 Clinical efficacy and safety

**FIGURE 3:**
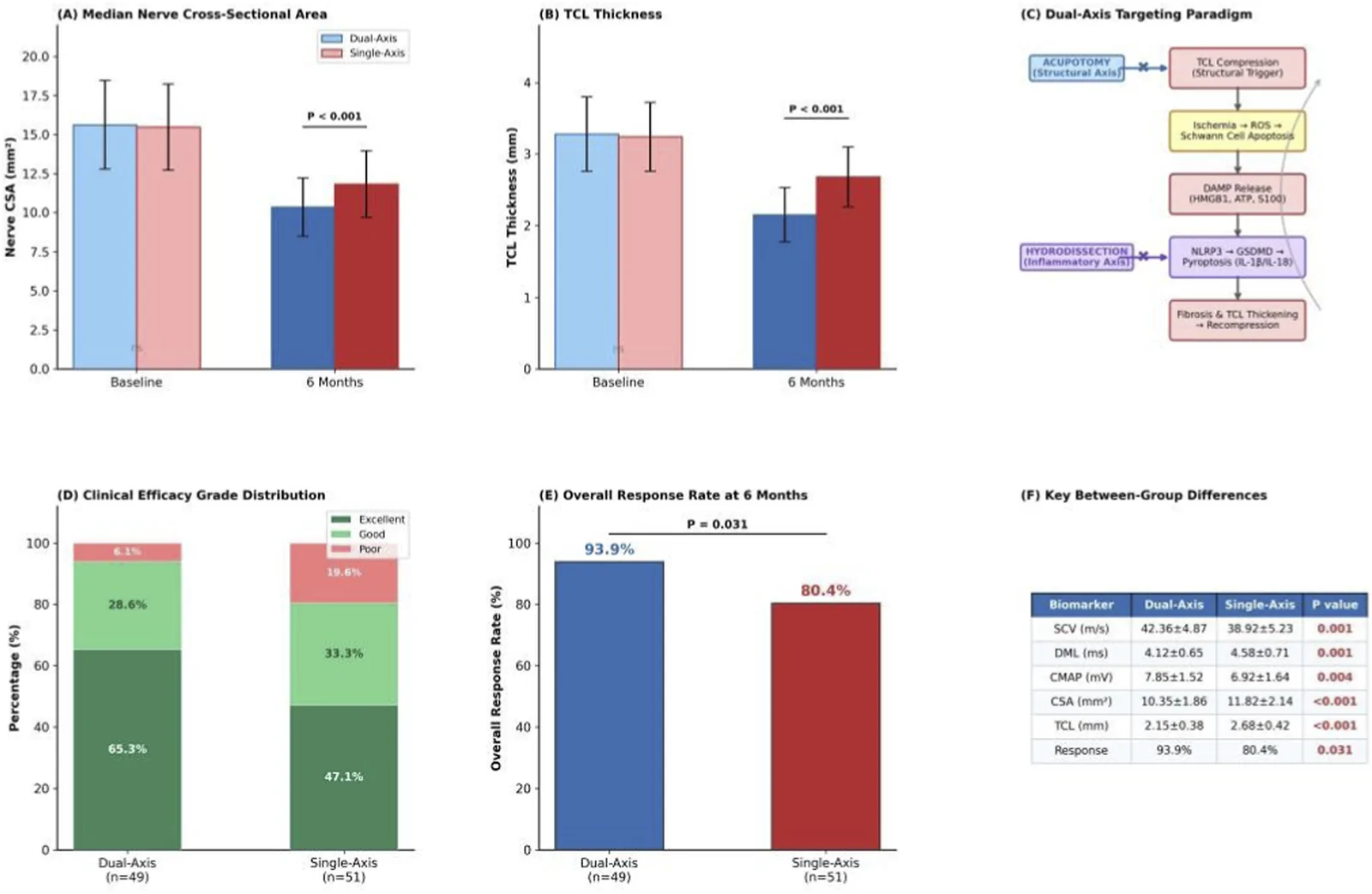
Ultrasonographic biomarkers, clinical efficacy, and dual-axis targeting paradigm .**(A)** Median nerve CSA and **(B)** TCL thickness at baseline and 6 months. **(C)** Schematic of the dual-axis targeting paradigm: acupotomy eliminates the structural–ischemic arm (TCL compression), while hydrodissection suppresses the inflammatory–pyroptotic arm (NLRP3–GSDMD–IL-1β cascade). **(D)** Clinical efficacy grade distribution. **(E)** Overall response rates. **(F)** Summary of key between-group differences at 6 months. Error bars: ±1 SD.

The overall response rate was significantly higher in the dual-axis group (93.9%, 46/49) than the single-axis group (80.4%, 41/51; Z = −2.156, P = 0.031; Table 7), with a higher proportion of “Excellent” outcomes (65.3% vs. 47.1%) (Figure 3).

**TABLE 7:** Clinical efficacy at 6 Months [n (%)].

Group	n	Excellent	Good	Poor	Response rate
Dual-axis	49	32 (65.3)	14 (28.6)	3 (6.1)	93.9%
Single-axis	51	24 (47.1)	17 (33.3)	10 (19.6)	80.4%

No serious adverse events occurred. Minor puncture-site hematomas: 2 cases (4.1%) dual-axis, 1 case (2.0%) single-axis, all self-resolving (P = 0.614, Fisher’s exact test).

## 4 Discussion

### 4.1 Cell death modalities in the CTS inflammatory microenvironment

The results demonstrate that dual-axis precision targeting of both the inflammatory and structural arms of the cell death–neuroinflammation cycle confers significantly superior outcomes compared with single-axis intervention. Schwann cells undergo intrinsic mitochondrial apoptosis under sustained compression, dismantling myelin sheaths and producing the electrophysiological hallmarks used as biomarkers in this study (Schmid et al., 2014; Gaudet et al., 2011). Beyond apoptosis, failure of cell clearance in the confined space likely triggers secondary necroptotic pathways resulting in massive DAMP release (Galluzzi et al., 2018; Weinlich et al., 2017).

Pyroptotic cell death represents a particularly significant amplification node. NLRP3 inflammasome assembly in synovial macrophages—driven by HMGB1 and ATP from dying Schwann cells—activates caspase-1, cleaving GSDMD to form membrane pores and processing pro-IL-1β and pro-IL-18 (Kelley et al., 2019; Shi et al., 2017; Yu et al., 2021). This pyroptotic cascade generates a self-sustaining inflammatory loop: IL-1β promotes fibrosis and TCL thickening, increasing intracarpal pressure and perpetuating ischemia, driving further apoptosis and DAMP release (Malakootian et al., 2023; Moalem-Taylor et al., 2017; Baričić et al., 2022). The interplay between apoptotic and pyroptotic pathways exemplifies the cross-talk between cell death modalities characteristic of chronic inflammatory disease (Bertheloot et al., 2021; Tang et al., 2019).

### 4.2 Hydrodissection: targeting the pyroptotic–inflammatory arm

Hydrodissection addresses the downstream inflammatory–pyroptotic arm through mechanical nerve–tissue separation and corticosteroid-mediated suppression of NF-κB, NLRP3 inflammasome priming, and pyroptotic IL-1β release (Ashworth et al., 2023; Kumar et al., 2023; Sveva et al., 2024). This explains the significant early improvements in both groups at 1 week. However, hydrodissection alone cannot abolish the structural ischemic driver; as corticosteroid effect wanes, persistent TCL compression reactivates the apoptotic cascade (Chan et al., 2025), reflected in the 80.4% response rate.

### 4.3 Acupotomy: eliminating the upstream ischemic cell death trigger

Acupotomy may substantially reduce intracarpal pressure through TCL release, potentially restoring endoneurial blood flow and normalizing mitochondrial homeostasis (Zhou et al., 2023; Chou et al., 2023; Sun et al., 2023). Attenuation of chronic ischemia may reduce the ROS generation that initiates intrinsic apoptotic signaling and lipid peroxidation (Malakootian et al., 2023; Gaudet et al., 2011). The sustained superiority of the dual-axis group from 1 month to 6 months is consistent with this structural intervention; however, the claim of permanent structural change is not substantiated by the 6-month data alone and requires longer-term follow-up to validate. The significantly greater TCL thickness reduction provides direct ultrasonographic confirmation of successful ligament release.

### 4.4 Electrophysiological and ultrasonographic parameters as functional biomarkers of cell death resolution

A central contribution of this study is characterizing routine clinical measurements as macro-level functional proxies of nerve recovery, consistent with the hypothesis of inflammatory cell death cascade attenuation—rather than as direct molecular biomarkers of specific cell death pathways. Superior electrophysiological recovery in the dual-axis group may reflect reduced Schwann cell apoptotic burden and enhanced remyelination (Koo et al., 2023), though this interpretation remains inferential without direct molecular assay data such as caspase-3/7 activity or TUNEL staining. The greater CSA reduction (10.35 ± 1.86 vs. 11.82 ± 2.14 mm^2^) is consistent with resolution of endoneurial edema potentially driven by IL-1β/TNF-α–mediated vascular permeability and DAMP-mediated fibrotic remodeling (Moalem-Taylor et al., 2017; Baričić et al., 2022; Savage and McKell, 2024; Guida et al., 2020). These clinically accessible parameters offer a practical monitoring framework without invasive tissue sampling; however, prospective studies incorporating serum cytokine panels and nerve biopsy immunohistochemistry are required to validate the proposed mechanistic interpretation.

#### 4.4.1 Likelihood of connection between electrophysiological/ultrasonographic features and neuroinflammatory processes

Although direct molecular measurement of neuroinflammatory mediators was not performed in this study, the observed pattern of electrophysiological and ultrasonographic changes is consistent with, and may plausibly reflect, underlying neuroinflammatory attenuation following combined treatment. Specifically, improvement in sensory conduction velocity (SCV) and reduction in distal motor latency (DML) are consistent with progressive remyelination, which is known to be impaired by pro-inflammatory cytokines—particularly TNF-α and IL-1β—that are elevated in the CTS microenvironment. The observed reduction in median nerve cross-sectional area (CSA) in the dual-axis group parallels the expected resolution of endoneurial edema, a hallmark of neuroinflammation mediated by IL-1β- and TNF-α-driven vascular permeability. The greater reduction in TCL thickness following acupotomy may reflect attenuation of the fibrotic remodeling driven by persistent neuroinflammatory stimuli. Taken together, these macro-level functional changes are consistent with a scenario in which dual-axis intervention more effectively attenuates neuroinflammatory activity than single-axis intervention alone—a hypothesis supported by the biological plausibility of the treatment mechanisms, even if not confirmed by direct biomarker measurement. Future studies incorporating serum inflammatory cytokine panels (e.g., IL-1β, TNF-α, IL-6) and nerve imaging indices at multiple time points would be valuable for evaluating this proposed mechanistic link more rigorously.

### 4.5 Implications for precision multi-target cell death modulation in inflammatory disease

The dual-axis paradigm—simultaneously targeting the inflammatory–pyroptotic arm and the structural–ischemic arm—aligns with the emerging consensus that single-pathway interventions are insufficient for conditions driven by cross-talking cell death modalities (Bertheloot et al., 2021; Tang et al., 2019). The failure of single-axis hydrodissection to maintain equivalent long-term outcomes illustrates the resilience of the “death–inflammation–redeath” cycle when only one node is targeted. This principle has broader implications for rheumatoid arthritis, inflammatory bowel disease, and other chronic inflammatory conditions where multi-node cell death modulation is increasingly advocated. The 93.9% vs. 80.4% response rate provides quantitative evidence for multi-target superiority.

### 4.6 Limitations and future directions

This study has several important limitations. First, the retrospective, non-randomized design introduces the retrospective, non-randomized design as the primary methodological threat: treatment allocation was not concealed, and unmeasured confounders—including dominant hand status, baseline nerve atrophy grade on ultrasound, specific symptom duration subgroups, and patient willingness to accept acupotomy—may have systematically favored the dual-axis group beyond what *post hoc* baseline comparisons can detect. These results should therefore be interpreted as hypothesis-generating rather than definitive. Second, the 6-month follow-up is insufficient to evaluate long-term recurrence, the durability of percutaneous TCL release, or potential late complications such as flexor tendon bowstringing; the existing surgical literature recommends a minimum of 12–24 months for meaningful recurrence data. Third, direct molecular quantification of cell death biomarkers—caspase-3/7 activity, GSDMD cleavage, circulating HMGB1, IL-1β/IL-18, or ferroptosis markers (4-HNE, GPX4)—was not performed; electrophysiological and ultrasonographic parameters should therefore be understood as macro-level functional proxies rather than direct molecular biomarkers. Fourth, the absence of a surgical control arm prevents comparison with open or endoscopic carpal tunnel release, which remains the standard of care for moderate-to-severe CTS refractory to conservative management; the dual-axis intervention should currently be positioned as a minimally invasive option for patients who are surgery-ineligible or who prefer non-surgical management. Fifth, inter-operator variability in acupotomy technique and TCL release completeness cannot be fully characterized retrospectively. Future prospective RCTs should incorporate concealed randomization, a surgical comparator arm, molecular cell death biomarker panels, extended follow-up (≥24 months), pre-specified operator competency standards, and tissue-level cell death analysis (TUNEL, caspase-1/GSDMD immunohistochemistry, RIPK3/MLKL expression) to validate these findings.

## 5 Conclusion

Ultrasound-guided hydrodissection combined with acupotomy achieves synergistic dual-axis disruption of the self-perpetuating “compression–ischemia–cell death–neuroinflammation” cycle in moderate-to-severe CTS. Corticosteroid hydrodissection may suppress the pyroptotic–inflammatory arm, while ultrasound-guided acupotomy may substantially attenuate the upstream ischemic stimulus for Schwann cell apoptotic signaling and DAMP release; however, direct molecular evidence for these proposed mechanisms was not collected in this study. Electrophysiological and ultrasonographic parameters serve as clinically accessible macro-level functional proxies of nerve recovery and neuroinflammatory attenuation. These findings are hypothesis-generating and require confirmation by prospective randomized trials incorporating surgical comparators, extended follow-up, and molecular biomarker validation before definitive clinical recommendations can be made.

## Data Availability

The datasets presented in this study can be found in online repositories. The names of the repository/repositories and accession number(s) can be found in the article/supplementary material.
